# Ocular immune responses, *Chlamydia trachomatis* infection and clinical signs of trachoma before and after azithromycin mass drug administration in a treatment naïve trachoma-endemic Tanzanian community

**DOI:** 10.1371/journal.pntd.0007559

**Published:** 2019-07-15

**Authors:** Athumani M. Ramadhani, Tamsyn Derrick, David Macleod, Patrick Massae, Aiweda Malisa, Kelvin Mbuya, Tara Mtuy, William Makupa, Chrissy H. Roberts, Robin L. Bailey, David C. W. Mabey, Martin J. Holland, Matthew J. Burton

**Affiliations:** 1 Clinical Research Department, Faculty of Infectious and Tropical Diseases, London School of Hygiene and Tropical Medicine, London, United Kingdom; 2 Kilimanjaro Christian Medical Centre, Moshi, Tanzania; 3 Department of Infectious Disease Epidemiology, Faculty of Epidemiology and Population Health, London School of Hygiene and Tropical Medicine, London, United Kingdom; RTI International, UNITED REPUBLIC OF TANZANIA

## Abstract

**Background:**

Trachoma, caused by *Chlamydia trachomatis*, remains the leading infectious cause of blindness worldwide. Persistence and progression of the resulting clinical disease appears to be an immunologically mediated process. Azithromycin, which is distributed at the community level for trachoma control, has immunomodulatory properties. We investigated the impact of one round of oral azithromycin on conjunctival immune responses, *C*. *trachomatis* infection and clinical signs three- and six- months post treatment relative to three pre-treatment time-points.

**Methodology:**

A cohort of children aged 6 to 10 years were recruited from a trachoma endemic region of northern Tanzania and were visited five times in a 12-month period. They were examined for clinical signs of trachoma and conjunctival swabs were collected for laboratory analysis. *C*. *trachomatis* infection was detected and the expression of 46 host genes was quantified using quantitative PCR. All community members were offered azithromycin treatment immediately after the six-month timepoint according to international guidelines.

**Findings:**

The prevalence of *C*. *trachomatis* infection and inflammatory disease signs were significantly reduced three- and six- months post-mass drug administration (MDA). *C*. *trachomatis* infection was strongly associated with clinical signs at all five time-points. A profound anti-inflammatory effect on conjunctival gene expression was observed 3 months post-MDA, however, gene expression had largely returned to pre-treatment levels of variation by 6 months. This effect was less marked, but still observed, after adjusting for *C*. *trachomatis* infection and when the analysis was restricted to individuals who were free from both infection and clinical disease at all five time-points. Interestingly, a modest effect was also observed in individuals who did not receive treatment.

**Conclusion:**

Conjunctival inflammation is the major clinical risk factor for progressive scarring trachoma, therefore, the reduction in inflammation associated with azithromycin treatment may be beneficial in limiting the development of potentially blinding disease sequelae. Future work should seek to determine whether this effect is mediated directly through inhibition of pro-inflammatory intracellular signalling molecules, through reductions in concurrent, sub-clinical infections, and/or through reduction of infection exposure.

## Author summary

Trachoma, caused by conjunctival infection with *Chlamydia trachomatis*, remains the leading infectious cause of blindness. Repeated infection during childhood can trigger prolonged inflammation, which is the main risk factor for conjunctival scarring. Azithromycin is distributed globally for trachoma control, however it is also widely reported to have immunomodulatory properties. This report investigated the impact of one round of oral azithromycin for trachoma control on conjunctival immune responses, clinical signs and *C*. *trachomatis* infection in Tanzanian children. A large anti-inflammatory effect of azithromycin on conjunctival gene expression was observed 3 months post-treatment, however, gene expression patterns had mostly resumed to pre-treatment levels by 6 months. The effect was evident after adjusting for *C*. *trachomatis* infection and when analysis was restricted to uninfected individuals, however it was also observed to a lesser extent in individuals that did not receive treatment. These findings suggest that azithromycin may have a direct immunomodulatory effect on conjunctival gene expression but that it may also reduce inflammation by reducing exposure to *C*. *trachomatis* and other infections. This anti-inflammatory effect could have therapeutic potential in limiting the development of disease sequelae, that goes beyond its effect on the clearance of ocular *C*. *trachomatis* infection.

## Introduction

Trachoma remains the leading infectious cause of blindness worldwide, with the greatest burden in sub-Saharan Africa [1]. Trachomatous disease is initiated by repeated conjunctival infection with C*hlamydia trachomatis*, which triggers prolonged inflammatory episodes that contribute to the development of conjunctival scarring [2]. Infection and clinical signs of active trachoma (follicular and papillary inflammation) are most frequently found in younger children [3]. Conjunctival scarring gradually accumulates through childhood, adolescence and into adult life. Eventually this results in the in-turning of the eyelid (entropion) and eyelashes (trichiasis), abrasion of the eyelashes against the cornea, severe visual impairment and blindness in later life. According to recent World Health Organisation (WHO) estimates, around 165.1 million people live in trachoma-endemic areas (of whom 89% are from WHO’s African region) [4] and 2.8 million have trichiasis [5]. The WHO advocates the use of the **SAFE** Strategy for trachoma control: **S**urgery to correct trichiasis, **A**ntibiotics to treat *C*. *trachomatis* infection, **F**acial cleanliness and **E**nvironmental improvements to suppress transmission [6].

Annual mass drug administration (MDA) with oral azithromycin for a minimum of 3 years is recommended for communities where the initial prevalence of the clinical sign *trachomatous inflammation-follicular* (TF) is ≥10% in children aged 1 to 9 years, with a recommended coverage of 80% of the whole community [7]. In low-prevalence settings this usually leads to a sustained reduction in *C*. *trachomatis* infection prevalence over time [8–11], however in highly endemic areas infection can re-emerge shortly after MDA [12].

Inflammatory disease signs are reported to persist longer than infection at both the individual and population levels, resulting in the observation of clinical signs in the absence of infection [13–16]. The correlation between clinical signs and *C*. *trachomatis* infection in communities prior to MDA is further reduced following treatment [14].

Previously we reported on the relationship between clinical signs, *C*. *trachomatis* infection and the expression of 91 immuno-fibrogenic and cell marker genes at the baseline time-point of a long-term cohort study of Tanzanian children [17]. We found an increase in transcripts related to Th1 and NK cell activity in individuals with *C*. *trachomatis* infection and an increase in matrix and fibrogenic factors in individuals with active disease in the absence of infection, supporting the findings of several earlier studies [16, 18–24]. However, the changes of these transcriptional responses in an untreated population and the changes that might occur following MDA with azithromycin have not previously been investigated.

Azithromycin is a macrolide antibiotic which has anti-inflammatory and immunomodulatory properties via inhibition of the transcription factor Nuclear Factor Kappa-B [25]. Azithromycin has been reported *in vitro* to suppress T-cell proliferation and activation and to reduce the expression of mucins and pro-inflammatory cytokines [26–28]. As a result azithromycin is found to be beneficial in the treatment of diseases characterised by pathological inflammation [29]. Azithromycin therefore has the potential to exert broad anti-inflammatory effects on conjunctival gene expression, independently of the clearance of *C*. *trachomatis*.

Here we investigate the changes in clinical signs of trachoma, *C*. *trachomatis* infection and host immune responses in a cohort of Tanzanian children three- and six-months post azithromycin MDA relative to three pre-treatment time-points. We also investigate the associations between clinical signs, infection and immune responses before and after MDA. This investigation uses data from the first five time-points of a four-year longitudinal study, the baseline findings of which have previously been reported [17].

## Methods

### Ethics statement

This study was reviewed and approved by the Ethics Committees of the Tanzania National Institute for Medical Research, Kilimanjaro Christian Medical University College and the London School of Hygiene & Tropical Medicine. The study adhered to the tenets of the Declaration of Helsinki. A field worker explained the nature of the study in detail in either Kiswahili or Maasai. Prior to enrolment of a child into this study, their parent or guardian provided written informed consent, on a consent form in Kiswahili, which was witnessed by a third person.

### Study design and population

This study was conducted in three adjacent trachoma endemic communities in Kilimanjaro and Arusha regions, Northern Tanzania. In January 2012 we recruited a cohort of children aged 6–10 years from these communities to study the pathogenesis of trachomatous conjunctival scarring. The cohort has subsequently been followed-up every three months for four years. All children aged 6–10 years, who were normally resident in the three villages, were eligible for inclusion. We chose this restricted age group as we considered that they were more likely to show evidence of incident or progressive conjunctival scarring during the four years of the study. The investigation presented in this paper is nested within this overall longitudinal study and uses data from the first five time-points only. The objectives of this nested investigation were to examine changes in *C*. *trachomatis* infection, clinical signs of trachoma and host immune responses, and the associations between them, three- and six-months post treatment relative to three pre-treatment time-points.

The study population and participant recruitment process are described in more detail in the report of baseline (time-point 1) findings [17]. In brief, these villages are relatively remote, geographically neighbours and have similar patterns of life and traditions. This area is predominately inhabited by people of the Maasai ethnic group. Pastoral activities are the main occupation. The area is dry for much of the year, except for the rainy season (February to May). Water supply is therefore limited, and largely depends on a long-distance water pipe scheme from Mount Kilimanjaro. Family units are organised in *Boma*, with living huts arranged in a circle around a central animal enclosure, which is often characterised by a high density of flies.

### Clinical assessments and sample collection

We visited the cohort of children every three months at their homes or schools. An experienced ophthalmic nurse examined their left eye for clinical signs of trachoma using x2.5 loupes and a bright torch. Signs were graded using the 1981 WHO ‘FPC’ detailed grading system [30]. This sub-divides the features into several four-point severity scales: follicles (F), papillary inflammation (P) and conjunctival scarring (C). This system corresponds to the WHO Simplified Trachoma Grading System in the following way: *Trachomatous inflammation-Follicular* (TF) is equivalent to F2/F3 and *Trachomatous inflammation-Intense* (TI) is equivalent to P3 [31]. Where we refer to “Active Trachoma”, we follow the widely used definition of TF (F2/3) and/or TI (P3). However for the purpose of this study, we also consider that both P2 and P3 represent clinically significant papillary inflammation, and refer to this as “TP” [18]. High resolution photographs (Nikon D90 camera with 105mm Macro lens) were taken of the examined eye for independent grading.

The conjunctiva of the left eye was anaesthetised with a drop of preservative-free proxymetacaine hydrochloride 0.5% w/v (Minims, Chauvin Pharmaceuticals Ltd, Surrey, UK). Two conjunctival swab samples (Dacron polyester, Puritan Medical Products Company, Maine, USA) were collected for *C*. *trachomatis* detection and gene expression analysis. The swabs were passed across the upper tarsal conjunctiva four times, with a quarter turn between each pass. The first swab was placed directly into a tube containing RNAlater solution (Thermo Fisher Scientific, Massachusetts, USA) and the second into a dry tube. The samples were placed into a cool box. Later the same day the dry swab samples were stored directly at -80°C and the RNAlater samples kept at 4–8°C overnight and then stored at -80°C.

### Trachoma control measures

The SAFE Strategy is being implemented in this region of Tanzania. Community members who had trachomatous trichiasis were offered free surgery in the local health facility. Azithromycin MDA was distributed to the members of the three villages by our field team under the auspices of the Tanzanian National NTD Control Programme in the Ministry of Health and with supervision by district eye coordinators. Azithromycin, donated to the National NTD Control Programme by Pfizer through the International Trachoma Initiative, was offered to all community members over the age of 6 months. Single-dose azithromycin MDA (1g for adults and 20mg/kg for children) was distributed annually for three years. For infants under six months, tetracycline eye ointment was provided to their primary carer to be applied twice a day to both eyes for six weeks. The project team provided repeated health education messages around hygiene and sanitation. The first round of MDA was administered to all individuals in the three cohort villages immediately after time-point 3. No adverse effects were reported.

### *C*. *trachomatis* detection

Two protocols were used for both genomic DNA extraction and *C*. *trachomatis* detection. For time-point 1 samples, genomic DNA was extracted from dry swabs using the PowerSoil DNA isolation Kit (MO Bio Laboratories, California, USA) according to manufacturer’s instructions. For time-points 2 to 5, genomic DNA was extracted from samples stored in RNAlater using the Norgen DNA/RNA Purification Kit (Norgen Biotek Corp, Canada) following the manufacturer’s instructions.

*C*. *trachomatis* was detected in the time-point 1 samples using a droplet digital PCR assay (ddPCR) and at time-points 2 to 5 using multiplex quantitative real-time PCR (qPCR) previously evaluated against ddPCR [17, 32, 33]. Both assays detect chlamydial plasmid open reading frame 2 (*pORF2*), *C*. *trachomatis* outer membrane complex protein B (*omcB*) and human endogenous control gene ribonuclease P/MRP Subunit P30 (*RPP30*) [33], using the same primer and probe sequences. The ddPCR reaction contained 5μl of DNA template and primers/probes at a final concentration of 0.3nM using Taqman mastermix. PCR reaction conditions were as follows: 95°C for 10 minutes, then 40 cycles of 95°C for 10 seconds and 60°C for 30 seconds and finally 98°C for 12 minutes. Droplets were then examined for fluorescence on a QX200TM Droplet Reader (Bio-Rad, UK), providing a quantitative result. The qPCR assay was performed on a ViiA7 thermal cycler (Thermo Fisher Scientific, Massachusetts, USA) using TaqMan Multiplex Master mix in a final volume of 20 μl, containing 4μl of DNA template and primers and probes each at a final concentration of 0.3nM. Cycling conditions were as follows: 95°C hold for 20 seconds followed by 40 cycles of 95°C for 1 second and 60°C for 20 seconds. Samples were tested in duplicate and were considered *C*. *trachomatis* positive if either replicate amplified *omcB* and/or *pORF2* with a cycle threshold (CT) value <40. In order to compare agreement between ddPCR and qPCR assays, Norgen-extracted DNA from time-point 2 samples (extracted from the first swab stored in RNAlater) were tested using both methods and the results are shown in S1A Table.

### Analysis of human gene expression

Total RNA was extracted from samples stored in RNAlater using the Norgen DNA/RNA Purification Kit (Norgen Biotek Corp, Canada) and reverse transcribed using the SuperScript VILO cDNA Synthesis Kit (Life Technologies) following the manufacturer’s instructions. Relative abundance of host gene targets was quantified by real-time PCR using customized TaqMan Microfluidic 384-well Array Cards (Thermo Fisher Scientific, Massachusetts, USA) on a ViiA7 real-time PCR machine (Thermo Fisher Scientific, Massachusetts, USA), as previously described [17]. A total of 46 genes of interest were selected based on our previously reported time-point 1 findings, in which we selected genes that were significantly associated with clinical signs and/or *C*. *trachomatis* infection status. *HPRT1* was included to each PCR run as an endogenous control gene.

### Statistical analysis

Data were managed in Microsoft Access. The ΔCT method was used to adjust for the concentration of input RNA by subtracting the cycle threshold (CT) value of each gene from the CT value of *HPRT1* in the same sample [34]. The distribution of ΔCT values were plotted to assess normality. Host gene expression, *C*. *trachomatis* infection and clinical data were analysed in STATA v14.

For each time-point the prevalence of clinical signs and *C*. *trachomatis* infection was estimated and the association between infection and each of TF and TP was estimated using logistic regression. The effect of MDA on infection, TF, TP and AT (Active Trachoma) was estimated using a random effects logistic regression. Each of infection, TF, TP and AT were used as the outcome variable in four separate regressions, the observations from the first three time-points were compared with the observations from time-point 4 (the first observation after the MDA) and participant ID was included as a random effect to account for the fact that these were repeated observations within individuals. An identical analysis was repeated comparing the observations from time-point 5 to those from before MDA to assess whether the estimated effect persisted at six months post treatment.

The change in mean ΔCT value from time-point 1 was plotted for 46 genes at each of the four subsequent time-points and inspected to identify any clear differences between time-points. The change in mean ΔCT following MDA was formally tested by comparing the mean ΔCT in the first three time-points with that from the fourth time-point using a random effects linear regression, with the ΔCT value of each gene as the outcome variable, whether an observation was before or after MDA as the exposure. Participant ID was included as a random effect to again account for repeated observations of the same individuals. An interaction term was included between before/after MDA and whether an individual was actually treated or not to assess the evidence of whether gene expression response after MDA was different in the treated and the untreated groups. These analyses were also repeated comparing the fifth time-point with the three pre-MDA time-points to identify if the effect persisted. Gene expression was then compared at time-point 4 only between treated and untreated individuals. These analyses were initially performed unadjusted and then adjusted for infection status (clinical signs were not adjusted for as they were likely to be caused by both infection and gene expression, rather than the other way around so adjusting for these could bias our estimates). The Benjamini and Hochberg method was used to control for the false discovery rate of 5% [35].

Multivariable linear regression was used at each of the five time-points presented in this report, to test the association of each gene’s expression with clinical signs and infection, adjusting for age and sex and assuming a false discovery rate (FDR) of 5% in multiple comparisons [35].

A Preferred Reporting Item (**STROBE_checklist_cohort 2-12-18**) is included in the supporting information. Accession numbers for each gene included in this analysis are included at the end of this manuscript. The protocols used in our analyses are accessible on protocols.io website using the accession number https://dx.doi.org/10.17504/protocols.io.zyhf7t6

### List of accession numbers for genes

HPRT1-Hs02800695, ALOX5-Hs01095330, CCL18-Hs00268113, CCL2-Hs00234140, CCL20-Hs01011368, CD247-Hs00609515, NCAM1-Hs00941830, CDH1-Hs01023894, CDH2-Hs00983056, CXCL13-Hs00757930, CXCL5-Hs01099660, DEFB4B;DEFB4A-Hs00175474, DUOX2-Hs00204187, FGF2-Hs00266645, IFNG-Hs00989291, IL10-Hs00961622, IL12B-Hs01011518, IL17A-Hs00174383, IL19-Hs00604657, IL1B-Hs01555410, IL21-Hs00222327, IL22-Hs01574154, IL23A-Hs00900828, IL6-Hs00985639, IL8-Hs00174103, MMP12-Hs00899662, MMP7-Hs01042796, MMP9-Hs00234579, MUC1-Hs00159357, MUC4-Hs00366414, MUC5AC-Hs00873651, MUC7-Hs03047182, MZB1-Hs00414907, NCR1-Hs00183118, PDGFB-Hs00966522, CD274-Hs01125301, S100A4-Hs00243202, S100A7-Hs01923188, SERPINB4;SERPINB3-Hs00741313, SOCS1-Hs00705164, SOCS3-Hs02330328, SPARCL1-Hs00949881, TGFB1-Hs00998133, VIM-Hs00185584, CTGF-Hs00170014, PTGS2-Hs00153133,

## Results

### Study participants

At census we registered a total of 666 children aged between 6 and 10 years who were eligible for recruitment at the beginning of this study from three trachoma-endemic villages. At time-point one 506 participants were assessed; their demography, clinical signs and infection status have previously reported in detail [17]. In general participants were predominantly from the Maasai ethnic group (652/666, 97.9%) with a similar number of males (332, [49.9%]) and females (334 [50.1%]) and a mean age of 7.01 years (SD 2.0) at the time of commencing the study. At time-points 2, 3, 4, and 5 we assessed 537, 466, 467 and 477 children, respectively. At each time-point some children were not examined due to being absent in the village, having moved away or declining to participate. After time-point 1, the recruitment of new participants into the longitudinal study was permitted at the second time-point only. MDA was offered immediately after time-point 3 to all members of the three cohort villages. The reported community-wide coverage was 68.7%. All study participants examined in time-point 3 (466) were treated except one who refused. At time-point 4, 392/466 (84.1%) of the individuals seen had been treated.

### Clinical signs of trachoma

At time-point 1 the clinical signs previously reported were based on grading of conjunctival photographs, to enable subsequent comparison with the final time-point for determination of scarring incidence and progression [17]. However, for consistency within this analysis of the first five time-points, the field grading data was used. The agreement between field and photograph grading for time-point 1 is shown in S1B Table. Kappa scores between field and photographs grading were 0.92 for TF and 0.68 for TP, with TP being slightly under-reported by field graders.

**Fig 1:**
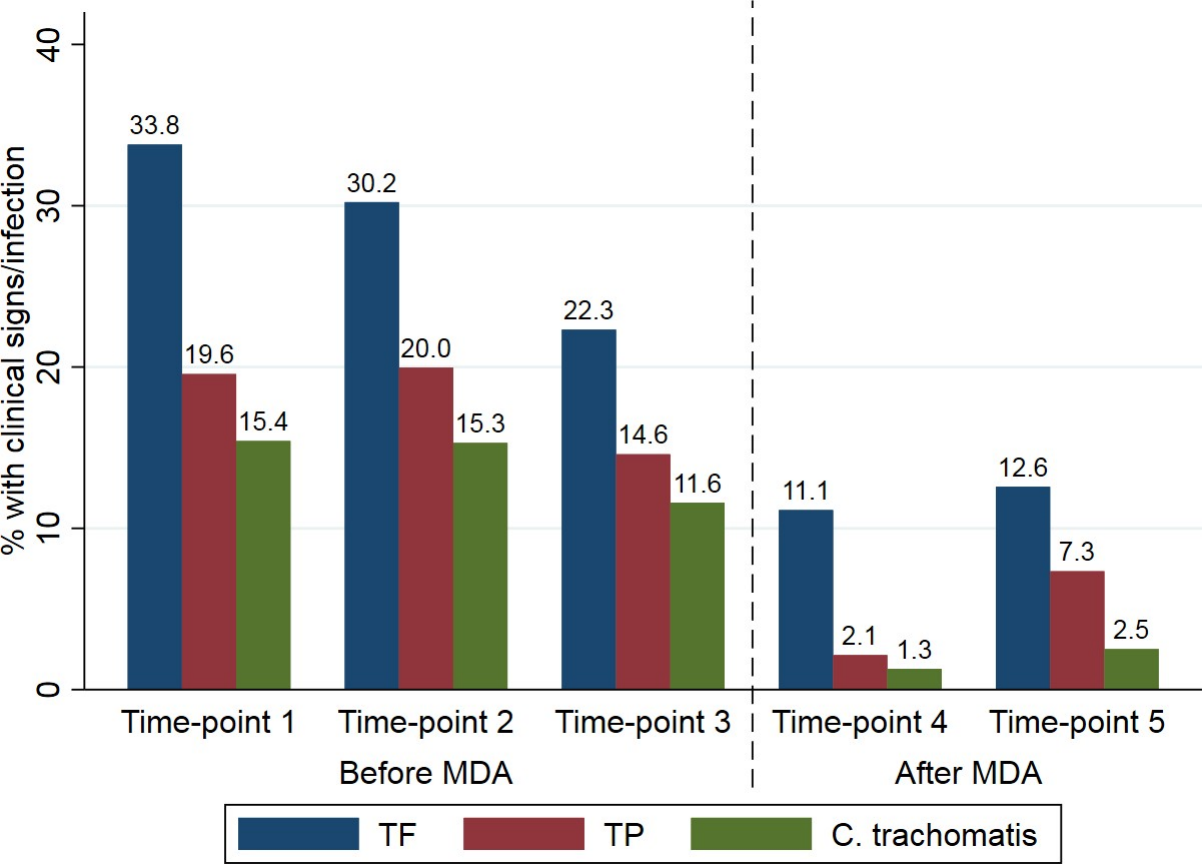
The prevalence of *C*. *trachomatis* infection and clinical signs before and after MDA. At time-points 1, 2, 3, 4, and 5 data are shown for 506, 537, 466, 467 and 477 children respectively.

The prevalence of TF in the first three time-points prior to MDA was fairly consistent (171/506 [33.8%], 163/537 [30.2%] and 104/467 [22.3%]), dropping to 52/467 (11.1%) and 61/479 (12.6%) post-MDA at time-points 4 and 5 (Fig 1). The prevalence of TP was consistently lower than TF and also dropped substantially following MDA (Fig 1). There were no statistically significant differences between males and females in terms of the proportion showing signs of TF and/or TP at any time-point (S2 Table), with the exception of time-point 1 where there was possibly a weak association between TP and female sex.

### *C*. *trachomatis* infection

**Fig 2:**
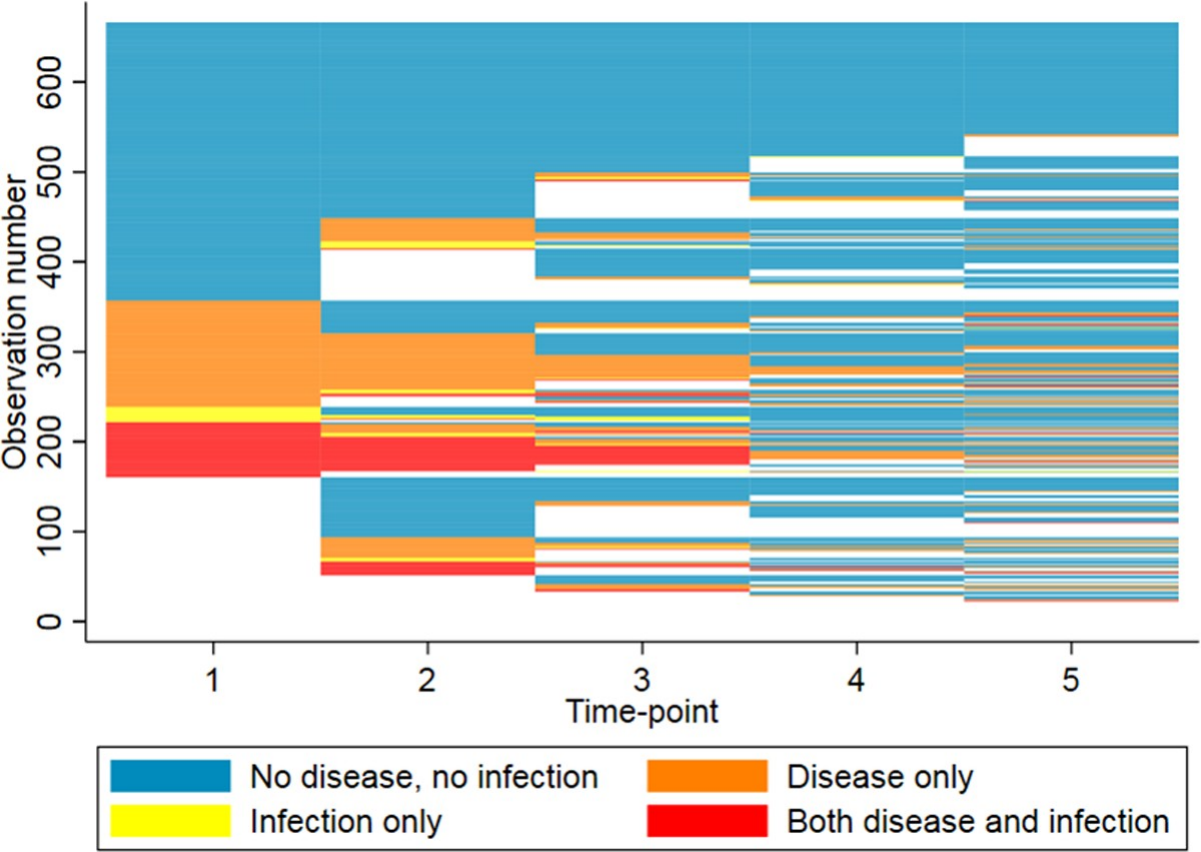
The clinical disease (Active Trachoma) and infection status of individuals at each time-point, ordered by status at baseline. Each row represents an individual and each column represents a time-point.

The prevalence of infection was fairly consistent prior to MDA, dropping very slightly from 15.4% and 15.3% at time-points 1 and 2 to 11.6% at time-point 3. Three months after azithromycin MDA (time-point 4) infection prevalence dropped to 1.3% and then increased slightly to 2.5% at time-point 5 (Fig 1). There was strong evidence for an association between *C*. *trachomatis* infection and clinical signs (TF, TP) at all five time-points (Table 1), with the exception of TP at time-point 5. There was a significant reduction in TF, TP and *C*. *trachomatis* detection in post-MDA time-points 4 and 5 relative to the combined odds at pre-MDA time-points 1–3 (Table 2). The inflammatory disease (TF and/or TI (active trachoma)) and infection status for each individual at each time-point is shown in Fig 2; participants were grouped by infection and disease status at baseline. There were no statistically significant differences between males and females in terms of the proportion testing positive for *C*. *trachomatis* at any time-point (S2 Table).

**Table 1:** The association between *C*. *trachomatis* detection and clinical signs by time-point. Clinical signs are based on field grading from all five time-points.

Time-point		Follicular Inflammation (TF)		Papillary inflammation (TP)	
		No TF (%)	TF (%)	No TP (%)	TP (%)
**1** **(n = 506)**	Ct infected (%)	18/335 (5.4)	60/171 (35.1)	38/407 (9.3)	40/99 (40.4)
	OR (95%CI)	-	9.52 (5.4–16.8)	-	6.58 (3.9–11.1)
	p-value	-	<0.0001	-	<0.0001
**2** **(n = 536)**	Ct infected (%)	29/374 (7.8)	53/162 (32.7)	38/429 (8.9)	44/107 (41.1)
	OR (95%CI)	-	5.78 (3.5–9.5)	-	7.19 (4.3–12.0)
	p-value	-	<0.0001	-	<0.0001
**3** **(n = 466)**	Ct infected (%)	19/362 (5.3)	35/104 (33.7)	30/398 (7.5)	24/68 (35.3)
	OR (95%CI)	-	9.16 (4.9–16.9)	-	6.69 (3.6–12.5)
	p-value	-	<0.0001	-	<0.0001
***Azithromycin MDA***					
**4** **(n = 467)**	Ct infected (%)	3/415 (0.7)	3/52 (5.8)	4/457 (0.9)	2/10 (20.0)
	OR (95%CI)	-	8.41 (1.7–42.8)	-	28.31 (4.5–177.5)
	p-value	-	0.010	-	<0.0001
**5** **(n = 477)**	Ct infected (%)	4/417 (1.0)	8/60 (13.1)	10/442 (2.3)	2/35 (5.7)
	OR (95%CI)	-	15.88 (4.6–54.5)	-	2.62 (0.6–12.4)
	p-value	-	<0.0001	-	0.226

**Table 2:** Comparison of *C*. *trachomatis* infection and disease signs between the pre-treatment (odds of time-points 1, 2 and 3) to the post-treatment time-points (4 and 5 analysed separately). Clinical signs are based on field grading from all five time-points.

		Infection	TF	TP	Active Trachoma
*Combined odds of pre-MDA time-points vs. time-point 4 (3 months post-MDA)*	OR	0.01	0.10	0.03	0.08
	(95%CI)	(0.004–0.04)	(0.06–0.17)	(0.01–0.07)	(0.05–0.13)
	p-value	<0.0001	<0.0001	<0.0001	<0.0001
*Combined odds of pre-MDA time-points vs. time-point 5 (6 months post-MDA)*	OR	0.04	0.15	0.18	0.14
	(95%CI)	(0.02–0.10)	(0.10–0.23)	(0.11–0.30)	(0.10–0.22)
	p-value	<0.0001	<0.0001	<0.0001	<0.0001

### Conjunctival gene expression

Forty-six genes of interest were quantified in all individuals who were sampled at each of the five time-points. All amplified targets were included in the analyses. For each time-point, multivariate linear regression models were constructed for expression of each gene to investigate associations with TF, TP and *C*. *trachomatis* infection, adjusting for age and sex (S3 Table). The associations between the expression of specific genes with clinical signs and *C*. *trachomatis* infection was similar at each time-point and was consistent with the baseline (time-point 1) report [17]. Briefly, in individuals with *C*. *trachomatis* infection, *IFNG*, *IL22*, *CCL2*, *IL12B*, *CD274*, *IL21*, *IL17A* and *SOCS1* genes were consistently the most upregulated and *S100A4*, *ALOX5*, *MMP7*, *MUC5AC*, *MUC7*, *MUC4*, *MUC1*, *CDH2* and *CDH1* genes were the most downregulated. In individuals with TF and TP, *S100A7*, *CCL18*, *MMP12*, *CXCL13*, *IL10*, *IL19*, *IL21* and *IL17A* were the most upregulated while *S100A4*, *SPARCL1*, *ALOX5* and *MUC5AC* were the most downregulated.

**Fig 3:**
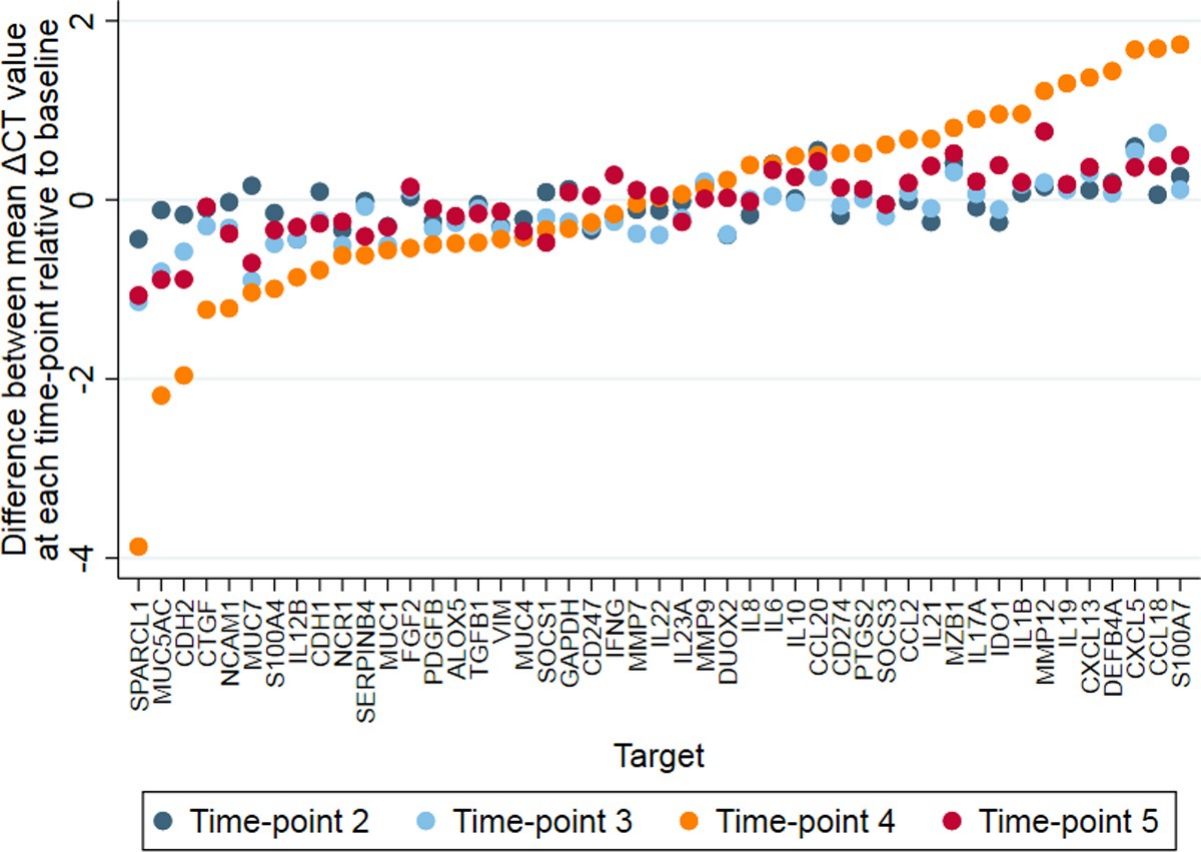
Variation in conjunctival gene expression. The difference in mean ΔCT value (across all individuals) for each gene at time-points 2, 3, 4 and 5 is shown relative to time-point 1. Values are adjusted for *C*. *trachomatis* infection and active trachoma and are ordered by difference in mean gene expression at Time-point 4.

For each target, the difference in mean gene expression (across all individuals) was calculated at each time-point relative to the mean expression at time-point 1 (Fig 3). There was only modest variability between these time-points, with the exception of time-point 4, three months after MDA, which showed marked differences compared to time-point 1 (and the other time-points). The largest increases in expression at time-point 4 relative to time-point 1 were found in *SPARCL1*, *MUC5AC*, *CDH2*, *CTGF*, *NCAM1*, *CDH1*, *MUC7*, *S100A4*, and *IL12B* (Fig 3). The largest decreases were in *S1007A*, *CCL18*, *CXCL5*, *DEFB4A*, *CXCL13*, *IL19*, *MMP12*, *IDO1*, *IL1B*, and *IL17A* (Fig 3). By time-point 5, six months after MDA, difference in mean gene expression had mostly returned to levels that were similar to those prior to treatment (Fig 3).

The change in mean ΔCT for the expression of each gene from the three time-points before MDA to the fourth time-point three months after MDA was estimated for all participants, and also separately for both individuals who received MDA and those who did not, adjusting for changes in *C*. *trachomatis* infection status (Table 3). This showed a significant change in mean ΔCT of most targets from before MDA compared with three months after MDA. Interestingly, this change was still observed in the untreated sub-group, albeit at a much reduced scale. The changes in mean ΔCT were larger when the analysis was not adjusted for *C*. *trachomatis* infection (S4 Table).

**Table 3:** Estimated Fold Changes (FC) with their respective p-values comparing the expression of each gene pre-MDA (time-points 1,2 and 3) and post-MDA (time-point 4). A FC of >1 indicates increased expression of the gene after MDA. Column 1 includes all participants, columns 2 and 3 show the results stratified by whether an individual actually received the treatment or not. The final column (p-value for interaction) assesses the evidence as to whether the fold change from before to after MDA is different in the treated and untreated groups. Results are ordered by FC of ‘‘**All**” individuals (column 1).

Target	All		Untreated Only		Treated Only		p-value for interaction
	FC	p-value	FC	p-value	FC	p-value	
SPARCL1	11.89	7.76 x10^-186	6.81	9.04 x10^-19	13.13	2.54 x10^-173	0.005
MUC5AC	4.02	3.95 x10^-145	2.89	1.20 x10^-14	4.26	5.35 x10^-136	0.009
CDH2	3.42	2.97 x10^-141	2.67	9.16 x10^-16	3.58	3.30 x10^-130	0.026
NCAM1	2.23	9.10 x10^-89	1.83	1.95 x10^-9	2.31	8.32 x10^-83	0.031
CTGF	2.20	3.57 x10^-96	2.25	1.27 x10^-17	2.20	4.10 x10^-81	0.818
MUC7	1.89	7.10 x10^-29	2.17	1.01 x10^-7	1.84	2.24 x10^-23	0.291
S100A4	1.82	7.83 x10^-79	1.75	1.56 x10^-12	1.83	5.50 x10^-69	0.612
CDH1	1.72	1.23 x10^-80	1.69	9.80 x10^-14	1.72	2.55 x10^-69	0.808
FGF2	1.50	2.86 x10^-9	1.48	0.019	1.50	4.19 x10^-8	0.934
SERPINB4	1.43	1.91 x10^-3	1.65	0.067	1.39	0.009	0.570
IL12B	1.42	5.47 x10^-15	1.49	4.10 x10^-4	1.41	1.97 x10^-12	0.657
TGFB1	1.34	2.53 x10^-45	1.43	4.80 x10^-12	1.33	6.55 x10^-36	0.178
ALOX5	1.29	7.49 x10^-39	1.37	7.71 x10^-11	1.27	1.24 x10^-30	0.133
MUC1	1.24	8.04 x10^-17	1.35	4.05 x10^-6	1.22	9.95 x10^-13	0.161
NCR1	1.23	2.51 x10^-10	1.25	0.007	1.23	7.76 x10^-9	0.835
PDGFB	1.22	7.96 x10^-14	1.35	1.26 x10^-5	1.20	2.59 x10^-10	0.130
GAPDH	1.20	5.05 x10^-11	1.49	1.68 x10^-8	1.16	1.33 x10^-6	0.001
SOCS1	1.20	2.64 x10^-8	1.49	5.69 x10^-7	1.15	6.66 x10^-5	0.003
MUC4	1.16	1.80 x10^-7	1.34	6.1 x10^-5	1.13	6.01 x10^-5	0.034
VIM	1.15	9.83 x10^-10	1.22	4.08 x10^-4	1.14	2.11 x10^-7	0.242
CD247	1.01	0.757	1.10	0.196	0.99	0.856	0.205
MMP9	0.95	0.242	0.98	0883	0.95	.0224	0.731
IFNG	0.95	0.219	0.93	0.521	0.95	0.293	0.860
MMP7	0.93	0.033	1.08	0.441	0.90	0.009	0.086
IL23A	0.88	5.88 x10^-4	1.00	0.989	0.86	1.73 x10^-4	0.127
IL22	0.83	0.020	1.26	0.232	0.76	0.002	0.016
IL6	0.83	5.24 x10^-4	0.83	0.150	0.83	0.001	0.959
CCL20	0.82	3.80 x10^-6	0.92	0.447	0.80	2.29 x10^-6	0.240
IL8	0.72	1.42 x10^-18	0.72	4.81 x10^-4	0.72	4.02 x10^-16	0.980
PTGS2	0.70	1.97 x10^-17	0.79	0.02.52	0.69	8.27 x10^-17	0.210
DUOX2	0.69	7.01 x10^-25	0.87	0.134	0.67	1.81 x10^-26	0.006
IL10	0.67	7.68 x10^-24	0.80	0.026	0.64	6.84 x10^-24	0.047
MZB1	0.65	3.20 x10^-17	0.71	0.008	0.64	6.23 x10^-16	0.466
CD274	0.63	6.37 x10^-37	0.77	0.004	0.61	1.28 x10^-36	0.018
CCL2	0.60	2.63 x10^-22	0.63	5.52 x10^-4	0.59	5.94 x10^-20	0.617
SOCS3	0.59	1.13 x10^-38	0.76	0.006	0.57	5.67 x10^-39	0.007
IL1B	0.52	1.78 x10^-43	0.61	2.81 x10^-5	0.50	1.55 x10^-40	0.140
IL21	0.51	3.18 x10^-24	0.72	0.044	0.48	1.03 x10^-24	0.022
IL17A	0.48	6.01 x10^-44	0.64	7.36 x10^-4	0.46	2.57 x10^-43	0.017
IDO1	0.44	6.25 x10^-90	0.68	1.69 x10^-4	0.41	4.74 x10^-93	9.55 x10^-6
MMP12	0.43	9.98 x10^-70	0.56	1.87 x10^-6	0.41	6.82 x10^-67	0.018
IL19	0.40	5.00 x10^-63	0.55	1.42 x10^-5	0.37	4.32 x10^-61	0.012
CXCL5	0.39	2.79 x10^-65	0.61	3.88 x10^-4	0.36	6.18 x10^-66	7.64 x10^-4
DEFB4A	0.38	1.32 x10^-64	0.52	1.16 x10^-5	0.36	1.94 x10^-62	0.015
CXCL13	0.38	1.19 x10^-47	0.50	3.58 x10^-5	0.36	3.28 x10^-45	0.067
CCL18	0.33	8.10 x10^-49	0.47	5.75 x10^-5	0.31	7.95 x10^-47	0.032
S100A7	0.28	4.90 x10^-55	0.53	0.002	0.25	3.61 x10^-56	7.03 x10^-4

To investigate the differences between the treated and untreated groups further, mean ΔCT were compared between the groups at time-point 4 only (S5 Table). This revealed only subtle differences in mean ΔCT between treated and untreated individuals. The anti-inflammatory effect of MDA on gene expression was observed even in individuals without any detectable episodes of chlamydial infection or clinical disease (F0, P0) at any of these five time-points (S6 Table).

## Discussion

### Infection

The prevalence of *C*. *trachomatis* was similar across the three time-points before MDA, suggesting that the infection prevalence was relatively stable in this antibiotic-naïve community at around 11% - 16%. The drop in the prevalence of infection and clinical signs at time-point 3 may have been due to medium term natural variation in the prevalence, as strains of *Chlamydia trachomatis* come and go, due to the introduction of public health education to the communities by the field team or possibly due to the change in seasons. There was a substantial reduction in infection prevalence at three months post-MDA, however, it rose slightly by six months post-MDA, suggesting some limited re-emergence of infection. This may be due to insufficient MDA coverage within the community, contact with individuals from surrounding untreated communities, or failure to complete the 6-week daily treatment course of tetracycline eye ointment for infants under 6 months. Members of these communities travel quite frequently to search for pastures and water for livestock, to visit markets and for social interactions with other communities. As a result, it was difficult to achieve high MDA coverage. Previous studies from Tanzania and The Gambia have also reported on the importance of contact between communities as a risk factor for reinfection following treatment [10, 11, 36].

### Disease

Clinical signs of inflammation were strongly correlated with *C*. *trachomatis* infection at all five time-points. In our previous systematic review and meta-analysis we found a strong correlation between TF and *C*. *trachomatis* infection and a moderate correlation between intense papillary inflammation (TI) and infection prior to initiation of MDA, however after treatment the correlation was weaker for TF and no correlation was found for TI [14]. Most of these earlier studies included multiple rounds of MDA and reported data several years after initiating treatment, therefore it might be too early to see this trend in our cohort. There was no consistent difference in the prevalence of clinical signs of inflammation between males and females, with the exception of the first timepoint which showed a non-significant trend of more TP in females.

### Gene expression

Our findings in this study of the associations between host gene expression, *C*. *trachomatis* infection and clinical signs of inflammation were consistent with previous reports from ourselves and others [16, 18, 21, 22, 24, 37, 38]. Targets that were consistently associated with clinical signs (TF/TP) at all five time-points included antimicrobial peptides (*S100A7)*, pro-inflammatory cytokines and chemokines *(CCL18*, *CXCL13*, *IL10*, *IL19*, *IL21*, *IL17A)*, matrix modifiers *(MMP12* and *SPARCL1)*, epithelial-mesenchymal transition markers *(S100A4)*, microbiota responses (*ALOX5)* and mucins (*MUC5AC)*. Likewise, *C*. *trachomatis* infection was consistently associated with pro-inflammatory cytokines and chemokines *(IFNG*, *IL22*, *CCL2*, *IL12B*, *IL21*, *IL17A)*, regulators/signalling pathways *(SOCS1*, *CD274)*, *S100A4*, *ALOX5*, matrix modifiers *(MMP7*, *SPARCL1)* and mucins (*MUC7)*. We discussed the functions of these genes and their potential roles in the clearance of *C*. *trachomatis* infection and immunopathology in detail in our baseline paper [17]. The results at each of the subsequent time-points support the data from baseline, suggesting that strong *IFNG/IL12* responses are important in the clearance of infection, whilst Th17 cell associated cytokines and matrix factors are associated with both infection and the clinical inflammation which persists after infection has been cleared.

Large changes in gene expression were detected at time-point 4, three months after MDA with azithromycin, relative to the three time-points prior to MDA. This variation in gene expression largely returned to pre-MDA levels by time-point 5, six months post-MDA. Azithromycin appeared to have an anti-inflammatory effect on gene expression, reversing the direction of gene expression change usually associated with clinical signs and *C*. *trachomatis* infection. Genes normally downregulated in individuals with *C*. *trachomatis* infection and/or inflammation were upregulated post-MDA (*SPARCL1*, *MUC5AC*, *CDH2*, *CTGF*, *NCAM1*, *CDH1*, *S100A4*, *MUC7*, and *FGF2*), whilst genes normally upregulated (*S100A7*, *CCL18*, *CXCL5*, *CXCL13*, *IL19*, *IDO1*, *MMP12*, *IL17A*, *IL1B* and *IL21*) were strongly downregulated post-MDA. The effect was greatest when *C*. *trachomatis* infection was not adjusted for, however the effect was still large after adjustment for infection, suggesting that azithromycin has an immunomodulatory effect on gene expression that is independent of the concurrent reduction in infection. This effect was also seen in individuals without any episodes of *C*. *trachomatis* infection and clinical signs of inflammation across all 5 time-points, supporting this hypothesis. However, we cannot exclude the possibility that azithromycin treatment reduced ocular infections with other sub-clinical or mild inflammation-causing organisms in these individuals. Interestingly, a change in mean gene expression post-MDA was also observed in individuals who did not receive treatment. This could be due to a reduction in transmission and therefore exposure to *C*. *trachomatis* and/or other infectious organisms within the community as a whole.

Azithromycin has previously been reported to have anti-inflammatory effects in humans, animal and *in vitro* models, leading to improved clinical outcomes through a combined approach of clearing infection and reducing pathological host inflammatory responses [39]. One pre-surgical dose of azithromycin reduced the level of pro-inflammatory cytokines and chemokines detected in oral fluid 6 days following dental implant surgery relative to amoxicillin [40]. Relative to other non-macrolide antibiotics, azithromycin reduced levels of IL-6, IL-8, TNF-α and GM-CSF proteins in individuals with pneumonia and rhinovirus infections [41–43]. MMP9 expression was reduced in the airways of lung transplanted individuals treated with azithromycin between 3 and 6 months [44], and in an experimental laminectomy model in rats, azithromycin was associated with reduction of fibrosis and inflammatory cell density six weeks after administration [45]. Immunomodulatory effects of azithromycin are thought to be enhanced by its long half-life in tissue, lasting for several weeks [46, 47]. In addition to localised anti-inflammatory effects, one round of azithromycin, administered for trachoma control, was associated with a large reduction in infectious and all-cause childhood mortality [48]; a finding which was reinforced by a large multi-country placebo-controlled clinical trial [49]. Our findings of an immunomodulatory effect of azithromycin are therefore consistent with published evidence and suggest that MDA for trachoma control may have an additional protective effect through a systemic reduction in inflammation.

This study has several limitations. It was only feasible to sample one eye from study participants, thus only the left eye was examined and sampled throughout the longitudinal study. The age range of study participants was limited due to the study design of the overall longitudinal study, which this investigation was nested within. The method of *C*. *trachomatis* detection was changed after the first time-point, which could introduce inconsistencies between the infection results of the first relative to later time-points. Agreement between the two methods used was however deemed acceptable (S1A table). The infection loads of discrepant results were very low and at around the limit of detection. Given the large sample size and the use of three pre-MDA time-points, this variation is not expected to significantly alter the results or their interpretation.

### Conclusions

We present evidence that one round of oral azithromycin treatment exerted a strong anti-inflammatory effect on conjunctival gene expression, detectable three months following treatment but mostly returning to pre-MDA levels by six months. This effect was also observed in individuals without *C*. *trachomatis* infection and clinical signs of inflammation across all five time-points, indicating that the immunomodulatory effect was at least in part independent of the reduction of *C*. *trachomatis* infection. Interestingly, a reduced effect was also seen in individuals who did not receive treatment, which could reflect a community reduction in infection transmission and exposure. A limitation of this study is that we cannot determine whether this effect is mediated directly through inhibition of pro-inflammatory intracellular signalling molecules, through reductions in concurrent, sub-clinical infections, and/or through reduction of infection exposure, and future work should seek to understand these mechanisms. Conjunctival papillary inflammation is a significant risk factor for scarring progression [2], therefore the anti-inflammatory effect of azithromycin might have therapeutic potential in limiting the development of disease sequelae, that goes beyond its effect on the prevalence of ocular *C*. *trachomatis* infection.

## Supporting information

S1 Checklist STROBE checklist. (DOC)

S1 Table Table A) 2 x 2 table showing agreement between qPCR and ddPCR assays for C. trachomatis detection in DNA extracted from conjunctival swabs at time-point 2. Sensitivity = 82.6% (95% CI 72.8–89.9), Specificity = 96.7% (95% CI 96.7–99.4), NPV = 96.6% (95% CI 94.8–97.6%); PPV = 91.0% (95% CI 82.9–95.5%); Cohens Kappa = 0.84, Accuracy: overall probability that a sample will be correctly classified 95.8% (95% CI 93.7–97.4), for these samples at this prevalence (16.4%) with ddPCR as the reference standard. Table B) Agreement between field and photo grading at baseline (time-point 1) for follicular and papillary inflammation in the conjunctiva. Kappa scores between field and photographs grading were 0.92 for TF and 0.68 for TP.
(DOCX)

S2 Table The relationship between sex and (i) clinical signs (from field grading) and (ii) C. trachomatis infection of each of the 5 time-points. The number of individuals with each clinical phenotype or infection is shown as a proportion of the total number of males and females at each time-point. Associations between sex and clinical phenotypes or infection were tested using logistic regression.
(DOCX)

S3 Table Multivariable linear regression models for conjunctival gene expression associated with clinical signs, *C*. *trachomatis*, female sex and age. FC = fold change. Using the Benjamini and Hochberg approach to adjust for multiple comparisons, in order to control the false discovery rate <5% only tests with a p-value below 0.027 are considered statistically significant.
(XLSX)

S4 Table Estimated Fold Changes (FC) with their respective p-values comparing the expression of each gene between the combined first three time-points (time-points 1, 2 and 3) before MDA and time-point 4 (three months after MDA), not adjusted for *C*. *trachomatis* infection. A FC of >1 indicates increased expression of the gene at time-point 4. Random effects multivariable linear regression of all individuals (first panel), untreated only (second panel) and treated only (third panel). The final column (p-value for interaction) provides evidence as to whether the fold change from before to after MDA is different in the treated and untreated groups. Results are ordered by FC of ‘‘**All**” individuals. Benjamini and Hochberg approach was used to adjust for multiple comparisons, in order to control the false discovery rate <5%, only tests with a p-value <0.035 are considered statistically significant.
(DOCX)

S5 Table Estimated Fold Change (FC) with their respective p-values for the expression of each gene at time-point 4 only (three months post MDA), comparing MDA treated (after time-point 3) to untreated individuals. Multivariable linear regression of all individuals adjusted (first panel) and not adjusted (second panel) for *C*. *trachomatis* infection. Results are ordered by FC of adjusted data with infection. Benjamini and Hochberg approach was used to adjust for multiple comparisons, in order to control false discovery rate <5%, only tests with a p-value <0.009 are considered statistically significant.
(DOCX)

S6 Table Estimated fold changes (FC) with their respective p-values comparing the expression of each gene between the combined first three time-points (1, 2 and 3) before MDA and separately time-points 4 and 5 (three and six months following MDA treatment), in 122 individuals who were free from infection and disease (F0, P0) at all 5 time-points. Results are ordered by Fold Change (FC) in pre-MDA time-points vs. time-point 4. Benjamini and Hochberg approach was used to adjust for multiple comparisons, in order to control the false discovery rate <5%, only tests with a p-value <0.035 are considered statistically significant.
(DOCX)
