# Supplementary material for: Ocular immune responses, Chlamydia trachomatis infection and clinical signs of trachoma before and after azithromycin mass drug administration in a treatment naïve trachoma-endemic Tanzanian community
Source: PLoS Negl Trop Dis. 2019 Jul 15;13(7):e0007559. doi: 10.1371/journal.pntd.0007559 (PMC6658141; doi:10.1371/journal.pntd.0007559)
Supplement: S1 Table — Table A) 2 x 2 table showing agreement between qPCR and ddPCR assays for C. trachomatis detection in DNA extracted from conjunctival swabs at time-point 2. Sensitivity = 82.6% (95% CI 72.8–89.9), Specificity = 96.7% (95% CI 96.7–99.4), NPV = 96.6% (95% CI 94.8–97.6%); PPV = 91.0% (95% CI 82.9–95.5%); Cohens Kappa = 0.84, Accuracy: overall probability that a sample will be correctly classified 95.8% (95% CI 93.7–97.4), for these samples at this prevalence (16.4%) with ddPCR as the reference standard. Table B) Agreement between field and photo grading at baseline (time-point 1) for follicular and papillary inflammation in the conjunctiva. Kappa scores between field and photographs grading were 0.92 for TF and 0.68 for TP. (DOCX) [file pntd.0007559.s002.docx]

**Supplementary Table 1a**. 2 x 2 table showing agreement between qPCR and ddPCR assays for *C. trachomatis* detection in DNA extracted from conjunctival swabs at time-point 2.

|  |  | **ddPCR** | |  |
| --- | --- | --- | --- | --- |
|  |  | **+** | **-** | **Total** |
| **qPCR** | **+** | **71 (13.6%)** | **7 (1.3 %)** | **78** |
|  | **-** | **15 (2.9%)** | **430 (82.2%)** | **445** |
|  | **Total** | **86** | **437** | **523** |

Sensitivity = 82.6% (95% CI 72.8 – 89.9), Specificity = 96.7% (95% CI 96.7 -99.4), NPV = 96.6% (95% CI 94.8 – 97.6%); PPV = 91.0% (95% CI 82.9 – 95.5%); Cohens Kappa = 0.84, Accuracy: overall probability that a sample will be correctly classified 95.8% (95% CI 93.7 – 97.4),for these samples at this prevalence (16.4%) with ddPCR as the reference standard.

**Supplementary Table 1b**. Agreement between field and photo grading at baseline (time-point 1) for follicular and papillary inflammation in the conjunctiva. Kappa scores between field and photographs grading were 0.92 for TF and 0.68 for TP.

| **Field Grading** | **Photo Grading** | | | |
| --- | --- | --- | --- | --- |
|  | ***No TF (%)*** | ***TF (%)*** |  |  |
| ***No TF*** | 317 (94.63%) | 18 (5.37%) |  |  |
| ***TF*** | 19 (11.11%) | 152 (88.89%) |  |  |
|  |  |  |  |  |
|  | ***No TP (%)*** | ***TP (%)*** |  |  |
| ***No TP*** | 345 (84.77%) | 62 (15.23%) |  |  |
| ***TP*** | 1 (1.01%) | 98 (98.99%) |  |  |
|  |  |  |  |  |
|  | ***F0 (%)*** | ***F1 (%)*** | ***F2 (%)*** | ***F3 (%)*** |
| ***F0*** | 140 (96.55%) | 4 (2.76%) | 1 (0.69) | 0 (0.00%) |
| ***F1*** | 61 (32.11%) | 112 (58.95%) | 17 (8.95%) | 0 (0.00%) |
| ***F2*** | 0 (0.00%) | 16 (21.33%) | 49 (65.33%) | 10 (13.33%) |
| ***F3*** | 0 (0.00%) | 3 (3.13%) | 14 (14.58%) | 79 (82.29%) |
|  |  |  |  |  |
|  | ***P0 (%)*** | ***P1 (%)*** | ***P2 (%)*** | ***P3 (%)*** |
| ***P0*** | 173 (60.49%) | 99 (34.62%) | 13 (4.55%) | 1 (0.35%) |
| ***P1*** | 6 (4.96%) | 67 (55.37%) | 41 (33.88%) | 7 (5.79%) |
| ***P2*** | 1 (1.85%) | 0 (0.00%) | 38 (70.37%) | 15 (27.78%) |
| ***P3*** | 0 (0.00%) | 0 (0.00%) | 4 (8.89%) | 41 (91.11%) |
