# Supplementary material for: Ocular immune responses, Chlamydia trachomatis infection and clinical signs of trachoma before and after azithromycin mass drug administration in a treatment naïve trachoma-endemic Tanzanian community
Source: PLoS Negl Trop Dis. 2019 Jul 15;13(7):e0007559. doi: 10.1371/journal.pntd.0007559 (PMC6658141; doi:10.1371/journal.pntd.0007559)
Supplement: S2 Table — The relationship between sex and (i) clinical signs (from field grading) and (ii) C. trachomatis infection of each of the 5 time-points. The number of individuals with each clinical phenotype or infection is shown as a proportion of the total number of males and females at each time-point. Associations between sex and clinical phenotypes or infection were tested using logistic regression. (DOCX) [file pntd.0007559.s003.docx]

**Supplementary Table 2.** The relationship between sex and (i) clinical signs (from field grading) and (ii) *C. trachomatis* infection of each of the 5 time-points. The number of individuals with each clinical phenotype or infection is shown as a proportion of the total number of males and females at each time-point. Associations between sex and clinical phenotypes or infection were tested using logistic regression.

| **Time-point 1** | | | | **Time-point 2** | | | | **Time-point 3** | | | | **MDA** | **Time-point 4** | | | | **Time-point 5** | | | |
| --- | --- | --- | --- | --- | --- | --- | --- | --- | --- | --- | --- | --- | --- | --- | --- | --- | --- | --- | --- | --- |
| **Male (%)** | **Female (%)** | **OR (95%CI)** | **p-**  **value** | **Male (%)** | **Female (%)** | **OR (95%CI)** | **p-value** | **Male (%)** | **Female (%)** | **OR (95%CI)** | **p-**  **value** |  | **Male (%)** | **Female (%)** | **OR (95%CI)** | **p-**  **value** | **Male (%)** | **Female (%)** | **OR (95%CI)** | **p-**  **value** |
| **Follicular Trachoma (TF)** | | | |  |  |  |  |  |  |  |  |  |  |  |  |  |  |  |  |  |
| 77/251 (30.7) | 94/255 (36.9) | 1.32  (0.9-1.9) | 0.142 | 72/258  (27.9) | 90/278  (32.4) | 1.24  (0.9-1.8) | 0.261 | 44/218  (20.2) | 60/248  (24.2) | 1.28  (0.8-2.0) | 0.278 |  | 23/216  (10.7) | 29/251  (11.6) | 1.10  (0.6-2.0) | 0.756 | 27/229  (11.8) | 34/248  (13.7) | 1.19  (0.7-2.0) | 0.531 |
| **Papillary Inflammation (TP)** | | | |  |  |  |  |  |  |  |  |  |  |  |  |  |  |  |  |  |
| 41/251 (16.3) | 58/255 (26.8) | 1.51  (1.0-2.4) | 0.070 | 45/258  (17.4) | 63/278  (22.7) | 1.39  (0.9-2.1) | 0.133 | 28/218  (12.8) | 41/248  (16.5) | 1.34  (0.8-2.3) | 0.264 |  | 6/216  (2.8) | 4/251 (1.6) | 0.57  (0.2-2.0) | 0.384 | 12/229 (5.2) | 23/248 (9.3) | 1.85  (0.9-3.8) | 0.096 |
| ***Chlamydia trachomatis*** | | | |  |  |  |  |  |  |  |  |  |  |  |  |  |  |  |  |  |
| 34/252 (13.5) | 44/255  (17.3) | 1.34  (0.8-2.2) | 0.241 | 32/258 (12.4) | 48/278  (17.3) | 1.47  (0.9-2.4) | 0.116 | 31/218  (14.2) | 24/248  (9.7) | 0.65  (0.4-1.1) | 0.131 |  | 1/216  (0.5) | 4/251 (1.60) | 3.48  (0.4-31) | 0.266 | 6/229 (2.6) | 6/248 (2.4) | 0.92  (0.3-2.9) | 0.889 |
