# Supplementary material for: Ocular immune responses, Chlamydia trachomatis infection and clinical signs of trachoma before and after azithromycin mass drug administration in a treatment naïve trachoma-endemic Tanzanian community
Source: PLoS Negl Trop Dis. 2019 Jul 15;13(7):e0007559. doi: 10.1371/journal.pntd.0007559 (PMC6658141; doi:10.1371/journal.pntd.0007559)
Supplement: S4 Table — A FC of >1 indicates increased expression of the gene at time-point 4. Random effects multivariable linear regression of all individuals (first panel), untreated only (second panel) and treated only (third panel). The final column (p-value for interaction) provides evidence as to whether the fold change from before to after MDA is different in the treated and untreated groups. Results are ordered by FC of ‘‘All” individuals. Benjamini and Hochberg approach was used to adjust for multiple comparisons, in order to control the false discovery rate <5%, only tests with a p-value <0.035 are considered statistically significant. (DOCX) [file pntd.0007559.s005.docx]

**Supplementary Table 4. Estimated Fold Changes (FC) with their respective p-values comparing the expression of each gene between the combined first three time-points (time-points 1, 2 and 3) before MDA and time-point 4 (three months after MDA), not adjusted for *C. trachomatis* infection.** A FC of >1 indicates increased expression of the gene at time-point 4. Random effects multivariable linear regression of all individuals (first panel), untreated only (second panel) and treated only (third panel). The final column (p-value for interaction) provides evidence as to whether the fold change from before to after MDA is different in the treated and untreated groups. Results are ordered by FC of ‘’**All**’’ individuals. Benjamini and Hochberg approach was used to adjust for multiple comparisons, in order to control the false discovery rate <5%, only tests with a p-value <0.035 are considered statistically significant.

| **Target** | **All** | | **Untreated Only** | | **Treated Only** | | **p-value for interaction** |
| --- | --- | --- | --- | --- | --- | --- | --- |
|  | **FC** | **p-value** | **FC** | **p-value** | **FC** | **p-value** |  |
| SPARCL1 | 18.65 | 2.75x10^-250 | 8.21 | 3.65x10^-23 | 21.77 | 1.32x10^-235 | 2.69X10^-5 |
| MUC5AC | 5.33 | 7.6x10^-215 | 3.14 | 3.06x10^-18 | 5.88 | 7.21x10^-206 | 1.19X10^-5 |
| CDH2 | 3.92 | 2.22x10^-169 | 2.72 | 3.72x10^-17 | 4.24 | 1.9x10^-158 | 6.84X10^-4 |
| MUC7 | 2.71 | 2.52x10^-72 | 2.45 | 9.19x10^-11 | 2.74 | 5.15x10^-62 | 0.458 |
| CTGF | 2.40 | 2.04x10^-118 | 2.29 | 1.12x10^-19 | 2.43 | 1.68x10^-101 | 0.553 |
| NCAM1 | 2.34 | 5.3x10^-90 | 1.79 | 8.89x10^-9 | 2.49 | 8.21x10^-86 | 0.003 |
| S100A4 | 2.30 | 1.87x10^-143 | 1.95 | 5.02x10^-17 | 2.37 | 4.66x10^-129 | 0.026 |
| CDH1 | 1.94 | 1.28x10^-122 | 1.78 | 1.38x10^-17 | 1.97 | 3.13x10^-107 | 0.173 |
| ALOX5 | 1.42 | 1.61x10^-65 | 1.45 | 1.37x10^-13 | 1.41 | 2.70x10^-53 | 0.606 |
| MUC1 | 1.39 | 7.87x10^-39 | 1.40 | 4.01x10^-8 | 1.40 | 2.12x10^-33 | 0.975 |
| FGF2 | 1.39 | 6.72X10^-6 | 1.41 | 0.046 | 1.42 | 1.77x10^-5 | 0.994 |
| TGFB1 | 1.34 | 9.68x10^-46 | 1.41 | 5.79x10^-12 | 1.32 | 2.41x10^-35 | 0.272 |
| MUC4 | 1.30 | 5.69x10^-19 | 1.40 | 2.54x10^-6 | 1.29 | 4.82x10^-15 | 0.273 |
| GAPDH | 1.29 | 6.09x10^-18 | 1.50 | 1.65x10^-8 | 1.25 | 1.06x10^-11 | 0.020 |
| IL12B | 1.24 | 7.69x10^-6 | 1.28 | 0.035 | 1.24 | 5.35x10^-5 | 0.794 |
| PDGFB | 1.22 | 1.49x10^-12 | 1.29 | 1.54x10^-4 | 1.20 | 1.01x10^-9 | 0.351 |
| NCR1 | 1.21 | 2.44x10^-8 | 1.15 | 0.086 | 1.22 | 6.90x10^-8 | 0.530 |
| VIM | 1.13 | 7.56x10^-8 | 1.17 | 0.005 | 1.13 | 3.59x10^-6 | 0.515 |
| SOCS1 | 1.13 | 2.42x10^-4 | 1.36 | 1.35x10^-4 | 1.09 | 0.021 | 0.012 |
| SX10RPINB4 | 1.13 | 0.341 | 1.34 | 0.320 | 1.09 | 0.565 | 0.520 |
| MMP7 | 1.11 | 0.006 | 1.17 | 0.086 | 1.09 | 0.028 | 0.487 |
| CD247 | 0.96 | 0.122 | 1.02 | 0.770 | 0.94 | 0.059 | 0.297 |
| MMP9 | 0.84 | 9.28x10^-5 | 0.91 | 0.371 | 0.83 | 1.37x10^-4 | 0.457 |
| IL23A | 0.83 | 2.24x10^-6 | 0.92 | 0.369 | 0.81 | 2.43x10^-6 | 0.250 |
| IL6 | 0.80 | 2.75x10^-5 | 0.77 | 0.050 | 0.81 | 3.65x10^-4 | 0.758 |
| CCL20 | 0.78 | 5.98x10^-8 | 0.88 | 0.263 | 0.76 | 8.80x10^-8 | 0.232 |
| IFNG | 0.76 | 7.28x10^-8 | 0.76 | 0.030 | 0.77 | 1.07x10^-6 | 0.985 |
| DUOX2 | 0.71 | 1.51x10^-22 | 0.87 | 0.116 | 0.69 | 1.05x10^-22 | 0.011 |
| PTGS2 | 0.68 | 1.96x10^-19 | 0.77 | 0.011 | 0.67 | 3.94x10^-18 | 0.203 |
| IL8 | 0.68 | 1.75x10^-24 | 0.69 | 7.73x10^-5 | 0.68 | 2.06x10^-20 | 0.822 |
| IL22 | 0.65 | 1.91x10^-6 | 0.95 | 0.801 | 0.60 | 2.79x10^-7 | 0.042 |
| IL10 | 0.60 | 7.40x10^-35 | 0.74 | 0.002 | 0.58 | 2.33x10^-33 | 0.029 |
| SOCS3 | 0.58 | 8.67x10^-40 | 0.73 | 0.001 | 0.56 | 3.03x10^-38 | 0.015 |
| MZB1 | 0.56 | 6.51x10^-26 | 0.65 | 0.001 | 0.55 | 7.23x10^-24 | 0.260 |
| CD274 | 0.54 | 1.82x10^-57 | 0.68 | 3.92x10^-5 | 0.51 | 4.37x10^-55 | 0.008 |
| CCL2 | 0.48 | 3.48x10^-37 | 0.55 | 1.23x10^-5 | 0.47 | 1.01x10^-32 | 0.341 |
| IL1B | 0.46 | 5.42x10^-59 | 0.56 | 7.91x10^-07 | 0.44 | 4.31x10^-54 | 0.054 |
| IDO1 | 0.41 | 7.77x10^-102 | 0.65 | 2.11x10^-5 | 0.38 | 3.28x10^-104 | 1.56X10^-6 |
| IL17A | 0.40 | 4.45x10^-62 | 0.57 | 2.46x10^-5 | 0.38 | 2.21x10^-59 | 0.005 |
| IL21 | 0.40 | 1.52x10^-39 | 0.61 | 0.003 | 0.37 | 6.53x10^-39 | 0.007 |
| MMP12 | 0.37 | 1.06x10^-87 | 0.51 | 4.56x10^-8 | 0.35 | 3.18x10^-82 | 0.005 |
| CXCL5 | 0.36 | 3.84x10^-72 | 0.59 | 2.11x10^-4 | 0.32 | 1.17x10^-72 | 1.04X10^-4 |
| DX10FB4A | 0.35 | 1.46x10^-64 | 0.50 | 4.51x10^-6 | 0.33 | 5.23x10^-61 | 0.013 |
| IL19 | 0.33 | 1.01x10^-79 | 0.48 | 2.57x10^-7 | 0.31 | 1.71x10^-75 | 0.005 |
| CXCL13 | 0.30 | 4.71x10^-66 | 0.44 | 9.38x10^-7 | 0.28 | 3.44x10^-62 | 0.015 |
| S100A7 | 0.24 | 2.33x10^-60 | 0.47 | 3.39x10^-4 | 0.21 | 3.46x10^-61 | 4.20X10^-4 |
| CCL18 | 0.23 | 1.32x10^-77 | 0.41 | 1.17x10^-6 | 0.21 | 9.46x10^-76 | 8.98X10^-4 |
