# Supplementary material for: Ocular immune responses, Chlamydia trachomatis infection and clinical signs of trachoma before and after azithromycin mass drug administration in a treatment naïve trachoma-endemic Tanzanian community
Source: PLoS Negl Trop Dis. 2019 Jul 15;13(7):e0007559. doi: 10.1371/journal.pntd.0007559 (PMC6658141; doi:10.1371/journal.pntd.0007559)
Supplement: S5 Table — Multivariable linear regression of all individuals adjusted (first panel) and not adjusted (second panel) for C. trachomatis infection. Results are ordered by FC of adjusted data with infection. Benjamini and Hochberg approach was used to adjust for multiple comparisons, in order to control false discovery rate <5%, only tests with a p-value <0.009 are considered statistically significant. (DOCX) [file pntd.0007559.s006.docx]

**Supplementary Table 5. Estimated Fold Change (FC) with their respective p-values for the expression of each gene at time-point 4 only (three months post MDA), comparing MDA treated (after time-point 3) to untreated individuals.** Multivariable linear regression of all individuals adjusted (first panel) and not adjusted (second panel) for *C. trachomatis* infection. Results are ordered by FC of adjusted data with infection. Benjamini and Hochberg approach was used to adjust for multiple comparisons, in order to control false discovery rate <5%, only tests with a p-value <0.009 are considered statistically significant.

| **Target** | **Adjusted for infection** | | **Not adjusted for infection** | |
| --- | --- | --- | --- | --- |
|  | **FC** | **p-value** | **FC** | **p-value** |
| SPARCL1 | 2.06 | 3.10x10^-04 | 2.34 | 2.55 x10^-5 |
| MUC5AC | 1.44 | 0.011 | 1.62 | 9.45 x10^-4 |
| MUC7 | 1.28 | 0.081 | 1.35 | 0.030 |
| CDH2 | 1.27 | 0.062 | 1.30 | 0.037 |
| NCAM1 | 1.19 | 0.055 | 1.19 | 0.048 |
| IFNG | 1.16 | 0.225 | 1.03 | 0.802 |
| S100A4 | 1.08 | 0.238 | 1.14 | 0.052 |
| MZB1 | 1.07 | 0.660 | 0.97 | 0.820 |
| NCR1 | 1.05 | 0.568 | 0.99 | 0.904 |
| CDH1 | 1.03 | 0.701 | 1.05 | 0.464 |
| CD247 | 1.02 | 0.748 | 0.97 | 0.668 |
| IL12B | 1.01 | 0.890 | 0.92 | 0.468 |
| VIM | 1.00 | 0.950 | 0.97 | 0.599 |
| TGFB1 | 0.98 | 0.738 | 0.96 | 0.429 |
| SERPINB4 | 0.97 | 0.913 | 0.89 | 0.700 |
| ALOX5 | 0.95 | 0.389 | 0.98 | 0.725 |
| CCL2 | 0.95 | 0.602 | 0.89 | 0.247 |
| PDGFB | 0.95 | 0.440 | 0.93 | 0.300 |
| IL6 | 0.94 | 0.627 | 0.91 | 0.479 |
| MMP9 | 0.94 | 0.552 | 0.90 | 0.331 |
| CTGF | 0.93 | 0.462 | 0.94 | 0.529 |
| IL23A | 0.93 | 0.340 | 0.88 | 0.110 |
| GAPDH | 0.92 | 0.330 | 0.91 | 0.275 |
| MMP7 | 0.91 | 0.370 | 0.93 | 0.520 |
| SOCS1 | 0.90 | 0.183 | 0.86 | 0.057 |
| MUC1 | 0.88 | 0.046 | 0.89 | 0.071 |
| IL8 | 0.88 | 0.208 | 0.88 | 0.203 |
| CD274 | 0.87 | 0.088 | 0.80 | 0.006 |
| CCL20 | 0.86 | 0.199 | 0.84 | 0.123 |
| PTGS2 | 0.85 | 0.100 | 0.84 | 0.083 |
| IL10 | 0.85 | 0.028 | 0.80 | 0.004 |
| IL1B | 0.84 | 0.083 | 0.81 | 0.034 |
| CXCL13 | 0.82 | 0.199 | 0.74 | 0.051 |
| MUC4 | 0.82 | 0.015 | 0.84 | 0.024 |
| FGF2 | 0.78 | 0.134 | 0.79 | 0.132 |
| SOCS3 | 0.78 | 0.008 | 0.76 | 0.003 |
| DUOX2 | 0.76 | 0.017 | 0.73 | 0.005 |
| IL19 | 0.74 | 0.018 | 0.69 | 0.003 |
| IL21 | 0.74 | 0.041 | 0.64 | 0.004 |
| CCL18 | 0.73 | 0.042 | 0.67 | 0.008 |
| IL17A | 0.73 | 0.013 | 0.66 | 0.001 |
| IL22 | 0.72 | 0.110 | 0.64 | 0.030 |
| MMP12 | 0.71 | 0.009 | 0.66 | 0.001 |
| DEFB4A | 0.71 | 0.068 | 0.69 | 0.042 |
| S100A7 | 0.70 | 0.030 | 0.63 | 0.005 |
| IDO1 | 0.64 | 8.99 x10^-4 | 0.59 | 1.03 x10^-4 |
| CXCL5 | 0.57 | 4.02E-04 | 0.56 | 1.98E-04 |
