# Supplementary material for: Ocular immune responses, Chlamydia trachomatis infection and clinical signs of trachoma before and after azithromycin mass drug administration in a treatment naïve trachoma-endemic Tanzanian community
Source: PLoS Negl Trop Dis. 2019 Jul 15;13(7):e0007559. doi: 10.1371/journal.pntd.0007559 (PMC6658141; doi:10.1371/journal.pntd.0007559)
Supplement: S6 Table — Results are ordered by Fold Change (FC) in pre-MDA time-points vs. time-point 4. Benjamini and Hochberg approach was used to adjust for multiple comparisons, in order to control the false discovery rate <5%, only tests with a p-value <0.035 are considered statistically significant. (DOCX) [file pntd.0007559.s007.docx]

**Supplementary Table 6. Estimated fold changes (FC) with their respective p-values comparing the expression of each gene between the combined first three time-points (1, 2 and 3) before MDA and separately time-points 4 and 5 (three and six months following MDA treatment), in 122 individuals who were free from infection and disease (F0, P0) at all 5 time-points.** Results are ordered by Fold Change (FC) in pre-MDA time-points vs. time-point 4. Benjamini and Hochberg approach was used to adjust for multiple comparisons, in order to control the false discovery rate <5%, only tests with a p-value <0.035 are considered statistically significant.

|  | **Pre-MDA vs time-point 4** | | **Pre-MDA vs time-point 5** | |
| --- | --- | --- | --- | --- |
| **Target** | **FC** | **p-value** | **FC** | **p-value** |
| SPARCL1 | 7.84 | 8.25x10^­-23^ | 1.44 | 0.130 |
| CDH2 | 3.34 | 7.15x10­^-21^ | 1.61 | 1.78x10^-04^ |
| MUC5AC | 3.07 | 1.75x10­^-16^ | 1.38 | 0.033 |
| CTGF | 1.99 | 2.71x10­^-10^ | 0.96 | 0.684 |
| NCAM1 | 1.93 | 4.07x10­^-15^ | 1.42 | 4.88x10^-05^ |
| CDH1 | 1.88 | 3.74x10­^-19^ | 1.17 | 0.019 |
| S100A4 | 1.74 | 3.57x10­^-14^ | 0.97 | 0.724 |
| MUC7 | 1.40 | 9.60x10­^-03^ | 1.03 | 0.846 |
| IL12B | 1.36 | 0.0147 | 1.01 | 0.927 |
| TGFB1 | 1.35 | 4.53x10­^-07^ | 1.10 | 0.111 |
| MUC1 | 1.29 | 8.18x10­^-05^ | 0.99 | 0.872 |
| ALOX5 | 1.29 | 8.31x10­^-09^ | 1.00 | 0.998 |
| PDGFB | 1.27 | 8.69x10­^-04^ | 0.94 | 0.346 |
| GAPDH | 1.25 | 2.23x10­^-04^ | 0.94 | 0.351 |
| SOCS1 | 1.23 | 0.0193 | 1.07 | 0.544 |
| IL23A | 1.16 | 0.13 | 1.04 | 0.694 |
| MUC4 | 1.12 | 0.139 | 0.91 | 0.359 |
| NCR1 | 1.09 | 0.355 | 0.92 | 0.416 |
| FGF2 | 1.08 | 0.683 | 0.97 | 0.890 |
| VIM | 1.07 | 0.26 | 0.90 | 0.102 |
| CD247 | 1.07 | 0.387 | 0.91 | 0.254 |
| MMP7 | 1.04 | 0.698 | 0.73 | 4.32x10^-04^ |
| IFNG | 0.93 | 0.534 | 0.82 | 0.125 |
| IL22 | 0.89 | 0.636 | 0.94 | 0.769 |
| CCL2 | 0.87 | 0.253 | 0.76 | 0.058 |
| SERPINB4 | 0.86 | 0.661 | 1.72 | 0.166 |
| MMP9 | 0.83 | 0.146 | 0.90 | 0.453 |
| DUOX2 | 0.83 | 0.049 | 0.61 | 5.06x10^-04^ |
| IL8 | 0.78 | 0.015 | 0.86 | 0.118 |
| CCL20 | 0.78 | 0.073 | 0.89 | 0.442 |
| PTGS2 | 0.75 | 0.022 | 0.82 | 0.123 |
| IL10 | 0.74 | 3.28x10­^-03^ | 0.83 | 0.061 |
| IL6 | 0.72 | 0.0384 | 0.77 | 0.079 |
| CD274 | 0.70 | 7.10x10­^-04^ | 0.83 | 0.092 |
| IL21 | 0.65 | 0.0166 | 0.71 | 0.064 |
| SOCS3 | 0.64 | 6.95x10­^-04^ | 0.76 | 0.057 |
| MZB1 | 0.61 | 3.29x10­^-04^ | 0.82 | 0.142 |
| IL17A | 0.60 | 8.87x10­^-04^ | 0.82 | 0.234 |
| IL1B | 0.58 | 5.77x10­^-05^ | 0.79 | 0.101 |
| IL19 | 0.56 | 4.06x10­^-05^ | 0.78 | 0.120 |
| IDO1 | 0.55 | 1.23x10­^-08^ | 0.69 | 2.28x10^-03^ |
| CCL18 | 0.53 | 3.07x10­^-04^ | 0.74 | 0.103 |
| CXCL13 | 0.48 | 1.64x10­^-05^ | 0.70 | 0.049 |
| CXCL5 | 0.46 | 4.97x10­^-07^ | 0.83 | 0.242 |
| MMP12 | 0.44 | 5.06x10­^-10^ | 0.54 | 7.17x10^-05^ |
| DEFB4A | 0.42 | 4.16x10­^-08^ | 0.64 | 0.012 |
| S100A7 | 0.41 | 6.23x10­^-08^ | 0.68 | 0.029 |
